# Protein Posttranslational Modifications: Roles in Aging and Age-Related Disease

**DOI:** 10.1155/2017/5716409

**Published:** 2017-08-15

**Authors:** Ana L. Santos, Ariel B. Lindner

**Affiliations:** Institut National de la Santé et de la Recherche Médicale, U1001, Université Paris Descartes and Sorbonne Paris Cité, Paris, France

## Abstract

Aging is characterized by the progressive decline of biochemical and physiological function in an individual. Consequently, aging is a major risk factor for diseases like cancer, obesity, and type 2 diabetes. The cellular and molecular mechanisms of aging are not well understood, nor is the relationship between aging and the onset of diseases. One of the hallmarks of aging is a decrease in cellular proteome homeostasis, allowing abnormal proteins to accumulate. This phenomenon is observed in both eukaryotes and prokaryotes, suggesting that the underlying molecular processes are evolutionarily conserved. Similar protein aggregation occurs in the pathogenesis of diseases like Alzheimer's and Parkinson's. Further, protein posttranslational modifications (PTMs), either spontaneous or physiological/pathological, are emerging as important markers of aging and aging-related diseases, though clear causality has not yet been firmly established. This review presents an overview of the interplay of PTMs in aging-associated molecular processes in eukaryotic aging models. Understanding PTM roles in aging could facilitate targeted therapies or interventions for age-related diseases. In addition, the study of PTMs in prokaryotes is highlighted, revealing the potential of simple prokaryotic models to uncover complex aging-associated molecular processes in the emerging field of microbiogerontology.

## 1. Introduction

In recent years, aging research has shifted its focus to the concept of healthspan, the extension of the period of life during which an individual remains healthy, rather than focusing only on ways to extend lifespan [1]. Worldwide, the average life expectancy at birth is now over sixty years as a result of improved healthcare access, decreased child mortality rates, reduced maternal mortality, improved lifestyle, and higher standards of living, among other factors [2]. This increase in life expectancy has led to a shift in population structure; between 2000 and 2050, the number of people aged 60 and over is expected to increase from 605 million to 2 billion worldwide [3]. A growing aged population can have a profound impact on society, both socially and economically [4]. Although a complete arrest of the aging process may be impossible, progress in developing pharmacological, dietary, and genetic interventions that lead to healthy aging might allow individuals to live longer while being less burdened by physical and/or mental decline. This issue is highlighted in the recent World Health Organization (WHO) Global Strategy and Action Plan on Ageing and Health (GSAP), which envisions a world in which everyone experiences healthy aging by maintaining the functional ability that enables well-being in old age [5].

From a biodemographic point of view, aging is defined as an exponential increase in mortality with time [6, 7], sometimes accompanied by a deceleration or plateau at later ages [6, 8–10]. Although the changes that underlie aging are complex [11], it is characterized by the gradual accumulation of a wide variety of molecular and cellular damage throughout the lifespan [12]. The nine proposed hallmarks of aging in mammals are genomic instability, telomere attrition, epigenetic alterations, loss of proteostasis, deregulated nutrient sensing, mitochondrial dysfunction, cellular senescence, stem cell exhaustion, and altered intercellular communication [13]. However, the connections between these hallmarks, their contributions to aging, and their links with frailty and disease remain incompletely understood [13]. In fact, uncovering the biological basis of aging is one of the greatest contemporary challenges in science [14].

Evidence suggests that it is possible to intervene at the level of the putative mechanisms underlying aging, subsequently leading to a slower rate of age-associated damage accumulation [12–14]. In this context, applying the multidisciplinary approaches of systems biology to probe the complex mechanisms of aging and various age-related disorders could generate the necessary evidence that leads to effective regulation of aging mechanisms [15]. Indeed, the use of model organisms has promoted rapid advances in the field of aging research through the identification of gene mutations that extend lifespan [16]. The major genes and pathways regulating lifespan are well conserved across eukaryotes such as yeast, worms, flies, and mammals [12].

Interestingly, epigenetics also plays a crucial role in aging [16–21]. While there are several different types of epigenetic mechanisms, protein posttranslational modifications (PTM) are intriguing contributors in regulating aging [22–27]. In this review, we discuss the involvement of PTMs in aging with a focus on PTM types, mechanisms of action, and detection methods. An overview of recent progress in PTMs and aging research across different model organisms is also included. Understanding PTMs and their contributions to aging provides a foundation for the development of interventions or targeted approaches to aging and age-related diseases.

## 2. Protein Posttranslational Modifications

Proteins are the basis of cellular and physiological functioning in living organisms, and the physical and chemical properties of proteins dictate their activities and functions. The primary sequence of a protein is a main determinant of protein folding and final conformation as well as biochemical activity, stability, and half-life [19]. However, at any given moment in the life of an individual, its proteome is up to two or three orders of magnitude more complex than the encoding genomes would predict [20]. One of the main routes of proteome expansion is through posttranslational modifications (PTM) of proteins. PTMs are present in both eukaryotes and prokaryotes, but it is estimated that PTMs are more common in eukaryotic cells, in which about 5% of the genome is dedicated to enzymes that carry out posttranslational modifications of proteins [20].

Protein PTM results from enzymatic or nonenzymatic attachment of specific chemical groups to amino acid side chains [20]. Such modifications occur either following protein translation or concomitant with translation. PTM influences both protein structure and physiological and cellular functions. Examples of enzymatic PTMs include phosphorylation, glycosylation, acetylation, methylation, sumoylation, palmitoylation, biotinylation, ubiquitination, nitration, chlorination, and oxidation/reduction [21]. Nonenzymatic PTMs include glycation, nitrosylation, oxidation/reduction, acetylation, and succination [22–26]. Some rare and unconventional PTMs, such as glypiation, neddylation, siderophorylation, AMPylation, and cholesteroylation, are also known to influence protein structure and function [27]. The major PTMs in eukaryotes, their target amino acid residue(s), and the enzyme(s) or protein(s) involved are shown in Table 1.

Alterations in the rate and extent of protein synthesis, accuracy, PTMs, and protein turnover are among the major molecular characteristics of aging. A decline in the cellular capacity to recognize and preferentially degrade the damaged proteins through proteasomal and lysosomal pathways ultimately leads to the accumulation of abnormal proteins during aging [28]. The consequent increase in molecular heterogeneity and impaired functioning of proteins is the basis of several age-related pathologies, including cataracts, sarcopenia, and various neurodegenerative diseases [29]. Therefore, understanding the spectrum of PTMs and their functional implications in aging will facilitate the development of effective intervention, prevention, and therapy for aging and age-related diseases.

Research on PTMs in prokaryotes started with the assumption that bacteria lack many features regularly found in more complex organisms. However, ongoing investigation continues to reveal new types of PTMs of bacterial proteins and their importance in bacterial adaptability and cell cycle control. Most bacterial PTMs are dynamic and reversible. This allows the cell to exploit them as regulatory devices. Among different bacterial PTMs, protein phosphorylation is the most extensively studied [30] and seems to be particularly relevant among important bacterial pathogens [31, 32]. Bacterial pathogens have developed diverse strategies to interact with host cells, manipulate their behaviors, and thus to survive and propagate. During pathogenesis, phosphorylation of proteins on hydroxyl amino acids (serine, threonine, and tyrosine) occurs at different stages, including cell-cell interaction and adherence, translocation of bacterial effectors into host cells, and changes in host cellular structure and function induced by infection. Among the various virulence factors involved in bacterial pathogenesis, special attention has been recently paid to the cell wall components, exopolysaccharides. A major breakthrough demonstrated the existence of a biological link between the activity of certain protein-tyrosine kinases/phosphatases and the production and/or transport of surface polysaccharides. From a general standpoint, the demonstration of a direct relationship between protein phosphorylation on serine/threonine/tyrosine and bacterial pathogenicity represents a novel concept with significance for deciphering the molecular and cellular mechanisms that underlie pathogenesis [33].

Moreover, several studies have begun uncovering the broad spectrum of PTMs involved in key bacterial cellular processes, including redox regulation via reversible S-thiolation [34], posttranslational hydroxylation [35], and the role of citrullination in the interaction between bacteria and human mucosal surfaces [36]. Several experimental studies have been done on the posttranslationally modified antimicrobial peptides known as lantibiotics [37]. Furthermore, studies have also pointed out an important role of PTMs on PII proteins, which are the key signal transduction proteins involved in the control of nitrogen metabolism in bacteria and archaea [38, 39]. More recently, Stannek et al. [40] have studied the regulatory mechanisms involving arginine phosphorylation and regulated proteolysis in *Bacillus subtilis* and proposed a mechanism whereby protein phosphorylation plays a role in quality control of bacterial proteins, targeting unstable and aggregation-prone proteins for degradation.

### 2.1. Methods to Detect Protein Posttranslational Modifications

Specific amino acid residues are subjected to PTMs depending on the chemistry of the reaction and the sequence specificity of the enzyme involved [20]. Initially, the detection of PTMs was carried out by various analytical methods, such as radiolabeling of the proteins, thin-layer chromatography, column chromatography, and/or polyacrylamide gel electrophoresis [31]. Other methods, such as protein sequencing by Edman degradation and Western blotting using protein-specific antibodies, have since been developed. Currently, antibody-based detection methods and mass spectrometry-based proteomic analysis are predominant methods used to detect and analyze PTMs. However, mass spectrometric methods are the only available tool to perform global or large-scale PTM analysis [32].

Antibody-based methods mostly rely on the availability of antibodies that can specifically recognize a modified amino acid residue within a protein or peptide. Such antibodies can be polyclonal or monoclonal and are developed against either the modified peptide/protein or against the modified amino acid. Moreover, antibody-based detection and quantification of PTMs on protein/peptide samples can be performed by two methods: chemiluminescence-based Western blotting and absorbance/fluorescence-based ELISA. However, the detection of PTMs depends entirely on the recognition site of the antibody used [41]. If the antibody detects only the modified amino acid, additional analysis—for instance, protein/peptide isolation and sequencing—should be performed to detect the sequence context of the modification. However, if the antibody detects the PTM within a specific sequence context, the presence of PTM at other sites will remain undetected.

Mass spectrometric detection of specific PTMs is based on mass changes [42]. Depending on the type of modification, a specific change in mass of the modified amino acid or peptide occurs. Subsequently, the change in mass is detected by the mass spectrometer to identify the presence of a PTM in a peptide sample. Using tandem mass spectrometric methods, identification of the specific site of PTM can be achieved by subsequent fragmentation and sequencing of the relevant peptide [43]. Yet, technical challenges hamper MS-based investigation of biologically important PTMs, such as ADP-ribosylation, one of the key signaling molecules that regulates DNA repair, a critical process in maintaining genome stability that is compromised in cancer and aging [38, 39].

Data increasingly implicate PTMs not only during aging and/or under pathological conditions but also for the normal functioning of the cell [39, 44]. In turn, PTMs are increasingly studied for their role in health and disease. For example, the precise and accurate measurement of distinct PTM-containing moieties offers potential biomarker utility to aid early diagnosis, prognosis, monitoring response to therapy and decisions regarding inclusion in clinical trials as new medicines are developed [45]. However, technical difficulties limit these studies, leaving many unanswered questions. The identification of unknown/unexpected PTMs by proteomic data reanalysis is an emerging subfield of proteomics recently boosted by the increased availability of raw data shared in public repositories. Notably, though, a sampling of the proteome in a given organism or cell provides only a snapshot of a highly dynamic process, confounding the analytical problem and ultimately arguing for time-resolved inventories [20]. Thus, while many tools are currently available for the study of PTMs, new methods are needed to further advance the study of these modifications.

## 3. PTMs in Aging

Generally, protein PTMs occur as a result of either modifying enzymes related to posttranslational processing (such as glycosylation) or signaling pathway activation (such as phosphorylation). Moreover, PTM patterns are known to be affected by disease conditions [46]. Similarly, the dysregulation of PTM is associated with the aging process [18, 47–49]. In this context, both enzymatic and nonenzymatic PTMs can undergo age-related alterations. Alteration in the pattern of nonenzymatic PTMs depends mainly on the nature of the modifying substances, such as metabolites and free radicals. For instance, reactive oxygen species can lead to oxidation of amino acid side chains (oxidation of thiols to different forms, oxidation of methionine, formation of carbonyl groups, etc.), modification by-products of glycoxidation and lipoxidation, and formation of protein-protein cross-links as well as oxidation of the protein backbone, resulting in protein fragmentation [50]. In contrast, changes in the nature of enzymatic PTMs rely primarily on the activities of modifying enzymes. In this review, we provide an overview of some of the most well-characterized PTMs implicated in aging and aging-associated pathologies across different levels of biological complexity.

### 3.1. A Brief Overview of Types of PTMs

**Scheme 1:**
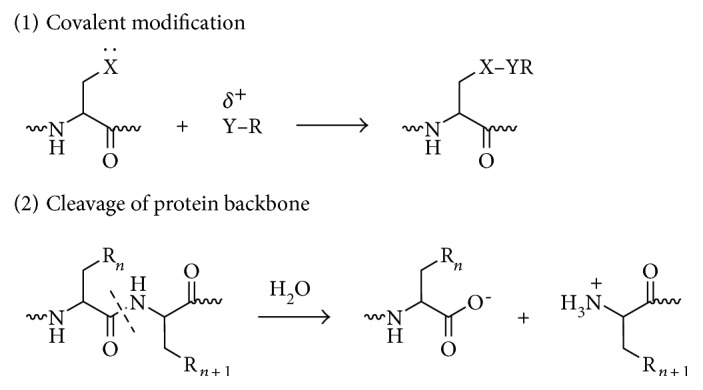
Two categories of posttranslational modifications of proteins: (1) covalent modification of a nucleophilic amino acid side chain by an electrophilic fragment of a cosubstrate and (2) cleavage of a protein backbone at a specific peptide bond. Reproduced with permission from Walsh et al. [20].

**Scheme 2:**
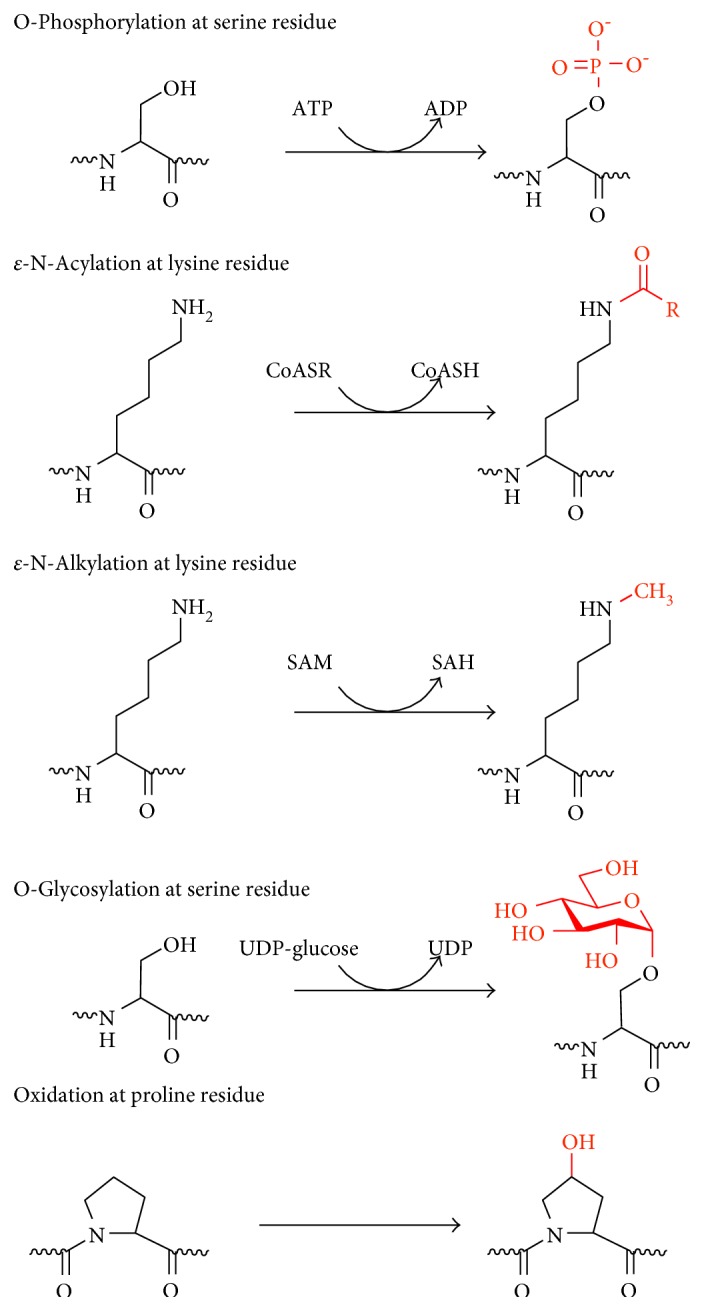
Five major types of covalent additions to protein side chains: phosphorylation, acylation, alkylation, glycosylation, and oxidation. Reproduced with permission from Walsh et al. [20].

Protein PTMs fall under two broad categories (Scheme 1). The first category encompasses covalent additions of some chemical group by enzymatic catalysis. Typically, an electrophilic fragment of a cosubstrate is added to an electron-rich protein side chain, which acts as a nucleophile in the transfer. The other category of PTMs encompasses covalent cleavage of peptide backbones. This cleavage occurs by one of two mechanisms: proteases or, less commonly, autocatalysis. Common covalent protein PTMs include phosphorylation, acylation, alkylation, glycosylation, and oxidation. These PTMs, catalyzed by dedicated mechanisms (Scheme 2), play roles in aging and age-related diseases. A brief description of the main types of PTMs associated with aging and age-related diseases is provided below.

#### 3.1.1. Protein Phosphorylation

The most common posttranslational modification, protein phosphorylation, is the reversible addition of a phosphoryl group from adenosine triphosphate (ATP) principally to serine, threonine, or tyrosine residues. This modification causes conformational changes that either (1) affect the catalytic activity to activate or inactive the protein and/or the tendency of a protein to misfold and aggregate [51] or (2) recruit other proteins to bind; both result in altered protein function and cell signaling [52]. Phosphorylated proteins have critical and well-known functions in diverse cellular processes across eukaryotes, but phosphorylation also occurs in prokaryotic cells. In humans, about one-third of proteins are estimated to be substrates for phosphorylation [53]. Indeed, phosphorylated proteins are now identified and characterized by high-throughput phosphoproteomics studies.

The reversibility of protein phosphorylation is attributed to the actions of kinases and phosphatases, which phosphorylate and dephosphorylate substrates, respectively. The temporal and spatial balance of kinase and phosphatase concentrations within a cell mediates the size of its phosphoproteome [54]. Accordingly, phosphatases have recently been proposed as potential next-generation therapeutic targets for age-related diseases, such as *α*-synucleinopathies like Parkinson's disease [55].

#### 3.1.2. Protein N-Acetylation

N-Acetylation is the reversible or irreversible transfer of an acetyl group to a nitrogen molecule through the actions of cleavage of methionine by methionine aminopeptidase (MAP) and the addition of an acetyl group from acetyl-CoA by N-acetyltransferase (NAT). Interestingly, 80–90% of eukaryotic proteins are acetylated, yet the underlying biological significance remains unclear [56]. In the case of histone proteins, which make up chromatin, lysine acetylation regulates gene transcription, thereby affecting the cell's transcriptome. Histone acetylation typically results in transcriptional activation; deacetylation typically results in transcriptional suppression. Acetylation occurs via histone acetyltransferases (HATs) and is reversible via the action of histone deacetylases (HDACs). One group of histone deacetylases are the sirtuins (silent information regulator), which maintain gene silencing via hypoacetylation. Sirtuins have been reported to aid in maintaining genomic stability [57].

Although first described in histones, acetylation is also observed in cytoplasmic proteins. Acetylated proteins can also be modulated by the cross-talk with other posttranslational modifications, including phosphorylation, ubiquitination, and methylation [58]. Therefore, acetylation may contribute to cell biology beyond transcriptional regulation [59].

#### 3.1.3. Protein Glycosylation

Protein glycosylation involves the addition of a diverse set of sugar moieties. This major type of PTM has significant implications for protein folding, conformation, distribution, stability, and activity. Glycosylated proteins can have additions of simple monosaccharides (e.g., nuclear transcription factors) or highly complex branched polysaccharides (e.g., cell surface receptors).

More than half of all mammalian proteins are believed to be glycosylated [60]. However, glycoprotein functions, at both molecular and cellular levels, remain unclear. While proteins exhibit improved stability and trafficking after glycosylation in vivo, glycan structures can alter protein functions or activities. These structures often result from the activities of glycan-processing enzymes working within a cell at any given time. However, the structures are sometimes protein-specific, depending on protein trafficking properties and interactions with other cellular factors [61].

There are three types of protein glycosylation in higher eukaryotes: N-linked, O-linked, and C-linked. These types reflect their glycosidic linkages to amino acid side chains [62]. In N-linked glycosylation, *β*-N-acetylglucosamine (GlcNAc) is attached through an amide linkage to the side chain of Asn in an AsnXaaSer/Thr group [63]. N-linked glycans have multiple functions. While they act as ligands for glycan-binding proteins in cell-cell communication, they also can regulate glycoprotein aggregation in the plasma membrane and affect the half-life of antibodies, cytokines, and hormones in serum [64].

O-linked glycosylation in higher eukaryotes occurs through several different mechanisms. The most abundant type of O-linked glycosylation is mucin-type, involved attachment of an *α*-N-acetylgalactosamine (GalNAc) to the hydroxyl group of Ser/Thr side chains [65, 66]. Aberrant expression of mucin-type O-linked glycans occurs in cancer cells [65] and may provide targets for anticancer vaccines [67].

O-linked glycosylation occurring with the addition of *α*-O-mannose is the only form of O-linked glycosylation in yeast but also occurs in the brains of higher eukaryotes [68, 69]. Higher eukaryotes also have an *α*-O-fucose modification of Ser/Thr residues that occur within the consensus sequon CysXaa_(3-5)_Ser = ThrCys [70]. This glycosylation modulates Notch signaling during eukaryotic development [71, 72]. Another modification, *β*-O-galactosylation, may contribute to rheumatoid arthritis [73–75].

Finally, C-linked glycosylation involves the addition of *α*-mannose (Man) to the 2-position of the indole side chain of tryptophan residues [76, 77]. While first identified on ribonuclease 2, it also occurs on other proteins, including MUC5AC and MUC5B [78], thrombospondin [79], and the Ebola virus soluble glycoprotein [80].

#### 3.1.4. Protein Ubiquitination and Sumoylation

Ubiquitination is the addition of an 8 kDa polypeptide to the N-terminus of target proteins via the C-terminal glycine of ubiquitin. The addition of one ubiquitin is followed by the formation of a ubiquitin polymer. The resultant polyubiquitinated proteins are recognized by the 26S proteasome in the protein degradation pathway [81].

Protein sumoylation is a reversible posttranslational modification whereby a small ubiquitin-like modifier (SUMO) is covalently attached to proteins [82]. Accordingly, protein sumoylation is mediated by a reversible enzymatic cascade in a manner similar to protein ubiquitination [83]. Like ubiquitin, SUMO is conjugated to the lysine side chains of target proteins via a cascade of activating, conjugating, and ligating enzymes, and it is removed by SUMO-specific isopeptidases [82]. Over the last few decades, it has been well established that sumoylation controls many aspects of nuclear function [84]. However, recent research has started to unveil a determinant role of protein sumoylation in many extranuclear neuronal processes and potentially in a wide range of neuropathological conditions [85].

#### 3.1.5. Protein S-Nitrosylation

Nitrosylation is a reversible addition of a nitric oxide (NO) to cysteine residues, forming S-nitrosothiols (SNOs), via redox-mediated reactions. S-Nitrosylation is used by cells to stabilize proteins, regulate gene expression, and provide NO donors. SNO generation, localization, activation, and catabolism are tightly regulated, and S-nitrosylation reactions depend on catalytic amounts of transition metals, O_2_, O_2_^−^, and pH, among other factors [86, 87]. Indeed, these molecules have a short half-life because of the action of enzymes like glutathione (GSH) and thioredoxin that denitrosylate proteins [88].

S-Nitrosylation is increasingly recognized as a ubiquitous regulatory reaction comparable to phosphorylation. SNOs may play an important role in many processes ranging from signal transduction, DNA repair, host defense, and blood pressure control to ion channel regulation and neurotransmission [89].

S-Nitrosylation specificity can mainly be achieved by two strategies. The existence of a consensus nitrosylation acid-based motif has been postulated [90]. S-Nitrosylation specificity may also be achieved through the subcellular localization of the NOSs, which may be in proximity to potential targets. The effect of NO on cells depends on its local concentration, the redox status of its immediate environment, and the susceptibility of target sites for modification [91]. Different degrees of accessibility to NO (RSNO) or different reaction rates with NO, as well as important functional differences in the -SH group being modified by NO, might explain why and how specific S-nitrosylation of precise cysteine residues induces protein modulation [92].

A classical example of SNOs is caspases, which mediate apoptosis. Stored in the mitochondrial intermembrane space as SNOs, caspases are then released into the cytoplasm and denitrosylated. The activated caspase then induces apoptosis [93].

#### 3.1.6. Protein Methylation

Alkyl substituents are attached to specific regions of proteins by PTM enzymes. The introduction of such alkyl groups results in the alteration of the hydrophobicity of the modified protein [94].

The most common type of protein alkylation is protein methylation. Methylation is a well-known PTM mediated by methyltransferases. One-carbon methyl groups are added to nitrogen or oxygen (N- and O-methylation, resp.) on amino acid side chains, increasing protein hydrophobicity or neutralizing a negative charge when bound to carboxylic acids. While N-methylation is irreversible, O-methylation is potentially reversible. Methylation occurs so often that its primary methyl donor, S-adenosyl methionine (SAM), is suggested as the most-used enzymatic substrate after ATP [56].

A common theme with methylated proteins, as is also the case with phosphorylated proteins, is the role this modification plays in the regulation of protein-protein interactions. For instance, the arginine methylation of proteins can either inhibit or promote protein-protein interactions depending on the type of methylation [95, 96].

Protein methylation has been most studied in the histones. The transfer of methyl groups from S-adenosyl methionine to histones is catalyzed by enzymes known as histone methyltransferases. The N-terminal tails of histones H3 and H4 receive methyl groups on specific lysines. Methylation then determines if gene transcription is activated or repressed, thus leading to different biological outcomes [97].

Histone methylation was traditionally thought to be irreversible. However, histone demethylases demonstrate the reversibility of this PTM [98]. Indeed, chromatin modification dynamic changes were imposed by an ability or inability to maintain equilibrium in the opposing effects of methylases and demethylases. The simultaneous removal of one histone methylation mark and an addition of another enable transcriptional tuning [99, 100].

Nonhistone proteins also exhibit methylation as a common PTM that regulates signal transduction via MAPK, WNT, BMP, Hippo, and JAK-STAT signaling pathways. Further, methylation works in concert with other types of PTMs, as well as with histone and nonhistone proteins, to exert influence on not only chromatin remodeling but also gene transcription, protein synthesis, and DNA repair [101].

#### 3.1.7. Protein Oxidation

The reaction of proteins with a variety of free radicals and reactive oxygen species (ROS) leads to oxidative protein modifications such as formation of protein hydroperoxides, hydroxylation of aromatic groups and aliphatic amino acid side chains, oxidation of sulfhydryl groups, oxidation of methionine residues, conversion of some amino acid residues into carbonyl groups, cleavage of the polypeptide chain, and formation of cross-linking bonds. Aromatic and sulfur-containing residues are particularly susceptible to oxidative modification [66–68].

Unless repaired or removed from cells, oxidized proteins are often toxic and can impair cellular viability [102], since oxidatively modified proteins can form large aggregates [103]. Oxidatively damaged proteins undergo selective proteolysis, primarily by the 20S proteasome in an ubiquitin- and ATP-independent way. Ultimately, upon extensive protein oxidation, these aggregates can become progressively resistant to proteolytic digestion and actually bind the 20S proteasome and irreversibly inhibit its activity [70–72].

Protein carbonylation is defined as an irreversible posttranslational modification (PTM) whereby a reactive carbonyl moiety, such as an aldehyde, ketone, or lactam, is introduced into a protein. The first identified source of protein-bound carbonyls was metal-catalyzed oxidation (MCO) [104]. MCO results from the Fenton reaction when transition metal ions are reduced in the presence of hydrogen peroxide, generating the highly reactive hydroxyl radicals in the process [105]. These hydroxyl radicals can oxidize amino acid side chains or cleave the protein backbone, leading to numerous modifications including reactive carbonyls [106]. For example, oxidation of proline and arginine results in the production of glutamic semialdehyde, while lysine is oxidized to aminoadipic semialdehyde and threonine to 2-amino-3-ketobutyric acid [107]. Direct oxidation of other amino acid residues can also lead to protein-bound carbonyls. Tryptophan oxidation by ROS produces at least seven oxidation products. Among them are kynurenine and N-formyl kynurenine, as well as their hydroxylated analogs, which contain aldehyde or keto groups formed by oxidative cleavage of the indole ring [108].

Another important source of protein-bound carbonyls is reactive lipid peroxidation products, which are produced during oxidation of polyunsaturated fatty acids [78–81]. Protein carbonylation can also occur via glycoxidation. Reactive *α*-carbonyls formed during glycoxidation, such as glyoxal, methylglyoxal, and 3-deoxyglucosone, can then modify the basic residues Lys and Arg to generate, for example, pyrralines and imidazolones [82, 83]. Glycation (i.e., the reaction of reducing sugars such as glucose or fructose with the side chains of lysine and arginine residues) forms Amadori and/or Hynes products. These glycated residues can be further decomposed by ROS into advanced glycation end products (AGE) carrying carbonylated moieties that can also contribute for protein carbonylation [109].

## 4. PTMs in Aging and Aging-Associated Diseases

Loss of cellular homeostasis during aging alters tissue functions, which leads to a general decline in physical/mental well-being and, ultimately, death. As individuals age, control of gene expression, which is orchestrated by multiple epigenetic factors, deteriorates. Epigenetic control of chromatin remodeling, through histone acetylation, is associated with cellular metabolism [110, 111]. Changes in metabolism with aging affect the concentration of acetyl-CoA and of citrate; this, in turn, alters the cytosolic level of acetyl-CoA. Altered acetyl-CoA levels, then, affect other metabolic processes such as the synthesis of fatty acids, exerting downstream effects on other physiological functions. Moreover, altered acetyl-CoA levels affect histone acetylation, thereby dysregulating transcription [110, 111]. These transcriptional changes occur with aging or with the progression of aging-related diseases. Acetylases and deacetylases likely exhibit different affinities for their acetyl-CoA and NAD^+^, respectively, which affects their responses to age-associated alterations in cofactor concentrations [112]. Thus, chromatin may act to sense changes in cellular metabolism [113]. In fact, lifespan can be extended by several manipulations that reverse age-dependent changes in chromatin structure, indicating the pivotal role of chromatin structure during aging [114]. Accordingly, mutations in genes that link metabolism and chromatin, such as lysine acetyltransferases (KATs), lysine deacetylases (KDACs) (sirtuins), and ATP citrate lyase (ACLY/ATPCL), can influence lifespan and the development of age-associated diseases [113].

Protein acetylation has been suggested to play a key role in the process of aging by enhancing the function of certain genes, most notably the AMPK regulatory subunit, which can promote longevity [115]. Likewise, it is widely accepted that sirtuins, a class of proteins that modulate stress responses and metabolism by removing the acetyl groups from target proteins, have an impact on lifespan and the aging process [116, 117]. Most notably, sirtuin SIRT3 plays a critical role in deacetylating many proteins in the mitochondria, suggesting that acetylation/deacetylation may be involved in the regulation of mitochondrial function [118]. More recently, it has been found that caloric restriction (CR), an intervention known to extend the lifespan in many organisms ranging from budding yeast to mammals, is associated with dramatic changes in mitochondrial acetylation. Many proteins are altered by acetylation in response to CR [119, 120]. These changes may contribute to mitochondrial adaptation to reduced caloric intake and may help to promote longevity. Likewise, regular exercise has been found to reduce oxidatively modified proteins in the brain with improved cognitive functions [121], through processes involving PTMs in histone tails controlled by HATs, HDACs, and histone demethylases [122].

Many pathways and processes appear to regulate the rate of aging and organismal susceptibility to age-related diseases such as neurodegeneration, atherosclerosis, and cancer. One process that is increasingly implicated is autophagy. First described in yeast, autophagy is a tightly regulated process stimulated by stressful conditions, such as starvation. Once activated, autophagy involves the recycling of old and damaged proteins and organelles to provide building blocks for new cellular components. Accordingly, disruption of this process results in diseased phenotypes and decreased lifespan, as revealed by studies using mouse models [96–98], *Caenorhabditis elegans*, *Drosophila melanogaster*, and *Saccharomyces cerevisiae* [90–93].

While the core components that regulate autophagy have been widely studied (e.g., [123]), less is known about the inputs that specifically alter this process, particularly how posttranslational modifications can influence autophagy flux and/or autophagic turnover. Three types of PTM, dubbed “The Three Musketeers of Autophagy”—phosphorylation, ubiquitylation, and acetylation—are crucial for autophagy induction, regulation, and fine-tuning and are influenced by a variety of stimuli. Understanding the mechanisms of autophagy regulation will provide biogerontologists deeper insight into the process and point to new therapeutic avenues [124].

### 4.1. Protein Oxidation and Aggregation

One of the earliest mentions of the effects of oxidative stress in cells can be found in a description of the chemical nature of pro-oxidant and antioxidant molecules [125]. A balance between oxidative and antioxidative effects maintains cellular health, whereas an imbalance is associated with diseases and aging. ROS are hallmarks of oxidative damage. The effects of an imbalanced redox status of cells primarily involve the modification of redox-sensitive molecules, such as the oxidation of cysteine and methionine in proteins, the peroxidation of lipids, and the oxidation of DNA bases [13]. The consequences of these modifications include direct effects on disease-causing proteins and indirect effects on enzymes and/or cofactors that in turn influence the function of disease-causing proteins [116, 117].

Several studies have identified proteins involved in mediating or countering reactive oxygen species production and action. A recent review [126] focusing on aging-related oxidative damage in the context of the damage accumulation theory of aging has stated that chronic oxidative damage is the primary cause of age-related diseases. Cellular senescence, defined as a loss of cell division, motility, and protein turnover, occurs as a result of damage accumulation over time and is considered an important feature of aging [13]. Morphological changes due to the accumulation of protein aggregates in the cells are also considered as a feature of cellular senescence induced by oxidative protein damage.

A wide range of aging-related diseases is at least in part associated with protein oxidative damage. These include eye diseases, metabolic disorders such as diabetes and obesity, inflammatory conditions such as arthritis, cardiovascular complications such as atherosclerosis, kidney disorders, respiratory disease, cancer, and neurodegenerative disorders such as Alzheimer's [119] and Parkinson's [120] diseases. Accordingly, Radman [127] recently proposed that aging and age-related diseases could be phenotypic consequences of proteome damage patterns.

Eye lens cataracts are a common affliction of aging populations that result in the progressive worsening of vision. One of the primary underlying changes during cataract formation is protein aggregation in the eye lens. While environmental factors like smoke, UV radiation, and chemical fumes contribute to the formation of cataracts, protein PTMs also play a significant role in the structure and stability of lens proteins, resulting in their aggregation within the lens [128]. Protein oxidation plays a particularly important role in lens protein aggregation, and antioxidants are often prescribed in the clinical management of cataracts [129]. Experimental studies using both human and mouse models have identified cysteine oxidation at the critical sites of several enzymes in human and mouse lens, including several metabolic enzymes, namely glyceraldehyde 3-phosphate dehydrogenase (GAPDH), glutathione synthase, aldehyde dehydrogenase, and sorbitol dehydrogenase, as well as protein deglycase DJ-1 (Parkinson disease protein 7 or PARK7) [130]. Extensive oxidation of intermediate filament proteins such as BFSP1 and BFSP12, vimentin, and cytokeratins, as well as the microfilament and microtubule filament proteins such as tubulin and actin, has also been reported [130].

Alzheimer's disease (AD) is one of the major aging-related disorders that severely impact the quality of life of elderly individuals [131]. The clinical symptoms of AD include a decline in cognitive function and memory and a state of confusion. At the cellular level, AD is associated primarily with two proteins: tau and amyloid-*β*. Dissociation of the microtubule-associated protein, tau, from the cytoskeleton in neuronal cells leads to its subsequent intracellular aggregation into paired helical filaments known as neurofibrillary tangles. Extracellular accumulation of amyloid-*β* peptide in the brain is another major factor driving the pathology of AD. The formation of amyloid-*β* peptide occurs due to the degradation of amyloid precursor protein (APP). Under normal circumstances, the peptide is degraded by proteases, including zinc proteases called neprilysins, endothelin-converting enzymes, and insulin-degrading enzyme [132]. Oxidative stress during aging may contribute to the inhibition of amyloid-degrading enzymes, which subsequently results in an aberrant extracellular accumulation of amyloid-*β* peptide in the brain [133].

The progression of AD is accompanied by hyperphosphorylation of tau. Hyperphosphorylated tau protein is found in degradation-resistant helical filament cores of neurofibrillary tangles [134]. Intriguingly, a recent report has shown that hydrogen peroxide-mediated oxidative stress can cause a temporary reduction in tau phosphorylation [135]. Further, 8-nitroguanosine 3′,5′-cyclic monophosphate (8-nitro-cGMP), a second messenger of the nitric oxide (NO) signaling pathway causing the oxidative S-guanylation of cysteine residues, results in a reduction of tau aggregation [136]. AD pathology involves posttranslationally modified forms of A*β* and tau, as well as other proteins. The study of these PTMs is key to the understanding of the molecular mechanisms associated with disease onset and also provides new opportunities for therapeutic strategies and drug development.

Parkinson's disease (PD) is another neurodegenerative disorder of unknown origin that affects approximately 6.3 million people worldwide [137]. The pathological hallmark lesions of PD are Lewy bodies (LBs), intraneuronal proteinaceous inclusions mainly comprising of misfolded *α*-synuclein [138]. LBs containing aggregated *α*-synuclein are found not only in PD but also in other neurodegenerative diseases, such as multiple system atrophy, dementia with LB, or AD [139]. Posttranslational modifications of *α*-synuclein, such as phosphorylation, ubiquitination, or nitration, are involved in the *α*-synuclein aggregation process and have different impacts on its cellular neurotoxicity [140–144].

The molecular mechanism involved in the clearance of *α*-synuclein aggregates is a central question for elucidating *α*-synuclein-related toxicity. However, clues to deciphering protein aggregation, which may eventually contribute to progress in understanding *α*-synucleinopathies, may emerge through the use of unicellular model organisms. Heterologous expression of *α*-synuclein in *Saccharomyces cerevisiae* also leads to protein aggregation and cellular toxicity characteristic of LB-containing human cells. In *S. cerevisiae*, cellular clearance mechanisms include ubiquitin-mediated 26S proteasome function as well as lysosome/vacuole-associated degradative pathways (i.e., autophagy). Various posttranslational modifications were found to change the cytotoxicity of *α*-synuclein and its distribution to different clearance pathways in *S. cerevisiae*. Several of the identified modification sites appear to be conserved from yeast to humans [145].

Aging is also a risk factor for cardiovascular diseases, such as hypertension, coronary heart disease, stroke, and heart failure. Several experimental and clinical observations support the hypothesis that excessive oxidative stress or reactive oxygen species (ROS) production plays a role in the pathogenesis of these diseases [146]. For instance, oxidative damage in cardiovascular disease is primarily related to low-density lipoproteins (LDL), which produce lipid peroxidation products such as lipid peroxides, isoprostanes, oxysterols, hydroxyl fatty acids, and aldehydes [147]. Likewise, recent studies on BMAL1 (brain and muscle ARNT-like protein-1) have shown that *Bmal1* null mice age prematurely because of increased ROS production. These mice also showed an aging-related decline in cardiac function, characterized by changes in ventricular diameter and ejection fraction [148]. Treatment with the antioxidant 4-hydroxy-2,2,6,6-tetramethylpiperidin-1-oxyl (TEMPOL) prevented compromised cardiac function in these mice. Protection of cardiac telomeres from the oxidation by TEMPOL in BMAL1-deficient mice was also observed, further supporting the therapeutic relevance of targeting protein oxidation in aging [148].

Studies of acute kidney injury and chronic kidney disease during aging have also highlighted the role of oxidatively damaged proteins and protein aggregates [149]. Proteins that are subjected to oxidative damage in the kidney include NADPH oxidase (NOX), heme oxygenase-1 (HO-1), thioredoxin 1 (TRX1), and the transient receptor potential cation channel, subfamily M, member 2 (TRPM2). In this context, a balance between oxidative stress and autophagy has been recognized as an important factor controlling inflammation and cell death in kidney disorders [150].

Various metabolic disorders, such as obesity, insulin resistance, and diabetes are characterized by increased body weight, high glucose levels, and reduced energy levels. Environmental and nutritional stresses are considered to be the main drivers of such metabolic disorders, potentially involving oxidative damage. The accumulation of reactive oxygen species mediates oxidative modification of metabolic enzymes and proteins, as does the consumption of high-carbohydrate or high-fat diets [151]. The enzyme methionine sulfoxide reductase A (MsrA) is an antioxidant enzyme in cells that is involved in countering the effects of oxidative stress and has been implicated significantly in developing protection against oxidative stress and protein maintenance, two crucial factors in the aging process [152]. A recent study [153] using transgenic mice has found that MsrA affects lifespan and ameliorates some of the effects of age-associated metabolic disorders, such as insulin resistance.

Taken together, these results highlight the role of protein oxidative damage in the process of aging and aging-related pathologies. Thus, pharmacological and nonpharmacological strategies that influence the oxidative stress balance of the cell are important as proximal strategies in the road towards extending healthspan.

### 4.2. Protein Chlorination in Aging

Reactive chlorine species are considered a primary source of enzymatically catalyzed protein chlorination [154]. The free-hydroxyl-containing tyrosine is the primary amino acid target for halogen modification. The enzyme myeloperoxidase catalyzes the formation of 3-chlorotyrosine [155]. An early study on protein chlorination [156] found that a tyrosine residue in apolipoprotein A-I (apoA-I) serves as a site for either chlorination or nitration depending on the action of either myeloperoxidase or peroxynitrite, respectively. Interestingly, chlorination but not nitration affected apoA-I function and markedly reduced its cholesterol efflux activity.

Elevated levels of myeloperoxidase are associated with chronic heart failure, and its expression increases in cardiac endothelial cells following exposure to hydrogen peroxide [157]. A recent study found that inhibition of myeloperoxidase using 2-thioxanthines resulted in a reduction of protein chlorination in a mouse model of peritonitis [158].

Skin aging is typically used as a physiological parameter to assess age-related changes in the body. A recent report on photoaging of the skin [159] proposed a link between inflammation-induced protein denitration and light-induced skin aging. The authors found elevated levels of halogenated tyrosine and inflammatory cells in skin samples both exposed to and protected from light, indicating that halogenation is likely a part of the normal aging process.

Neurodegeneration is another major consequence of aging that occurs due to a combination of factors, including oxidative stress. A recent report found that serotonin acts as a scavenger of hypochlorous acid (HOCl) in the brain [160] and prevents HOCl-induced oxidation of 2-thio-5-nitrobenzoate, loss of cellular *α*-ketoglutarate dehydrogenase activity, and cell death. Intriguingly, the biphasic removal of HOCl and subsequent prevention of 2-thio-5-nitrobenzoate oxidation involves HOCl-induced chlorination of serotonin as well as the formation of inactive aggregates of chlorinated serotonin, implicating a feedback process. Furthermore, selective serotonin reuptake inhibitors, such as fluoxetine, reduce protein chlorination in the brain, suggesting a potential therapeutic approach against age-related protein chlorination effects [160].

### 4.3. Protein Nitration

Nitration is an oxidative protein modification that occurs on tyrosine residues. Excess levels of reactive nitrogen species (RNS) are the primary source of nitrating reactions [154]. The excessive presence of ROS, along with RNS, leads to the formation of additional nitrating entities, namely peroxynitrite. One common example of RNS-induced protein nitration is the formation of 3-nitrotyrosine, which is associated with increased nitroxidative stress during the aging process [161]. Tyrosine nitration modifies the biochemical properties of the amino acid, including its pKa, redox potential, hydrophobicity, and size, subsequently leading to significant changes in the structure and function of affected proteins. Alterations in protein biochemistry provoke the cellular and physiological manifestations of nitration in aging. Additionally, protein tyrosine nitration is mediated by nonenzymatic free radical reactions involving the formation of an intermediate tyrosyl radical. Studies using fast reaction kinetics and bioanalytical methods as well as structural assessments using electron paramagnetic resonance have enabled the comprehensive characterization of tyrosine nitration [162]. Recent studies have shown that membrane-associated protein tyrosine nitration involves oxidation by lipid peroxyl radicals, a by-product of membrane lipid peroxidation, which is also associated with aging [163]. Moreover, several studies have revealed that protein tyrosine nitration occurs site-specifically to a few tyrosine residues within the target proteins and, thereby, is restricted to a fraction of the proteome [162]. The spatial and temporal localization of nitrating entities plays an important role in selecting the tyrosine residue within a target protein. Studies on mitochondrial proteins that are homogenously nitrated have further supported the site-specific selectivity as well as the overall effects of protein tyrosine nitration in aging and age-associated diseases [58, 59]. While protein tyrosine nitration was initially thought to be irreversible, recent studies have identified a denitrase enzyme [164]. Denitrase activity is found in a range of tissues and cells but not in smooth muscle cells. One recent study has demonstrated that denitrase utilizes nitrated cyclooxygease-1 (COX-1) as a substrate to facilitate the denitration reaction, suggesting that the reversible cycle of nitration and denitration may play a role in regulating cellular oxidative/nitrosative burdens, which subsequently modulate aging [164].

Another recent study [165] regarding the effect of protein nitration in age-related systemic inflammation (systemic inflammatory response syndrome or SIRS) has shown that toxemia-induced lung injury increases the level of protein tyrosine nitration and reduces the activity of superoxide dismutase in mouse lung. Additionally, aged mice showed higher protein nitration in the vascular endothelia compared to younger mice. The specific proteins that maintain pulmonary vascular permeability also showed higher tyrosine nitration, including profilin-1, transgelin-2, LASP 1, tropomyosin, and myosin [165].

## 5. Bacteria as Potential Simple Tools to Study PTMs in Aging and Age-Associated Pathologies

**Figure 1:**
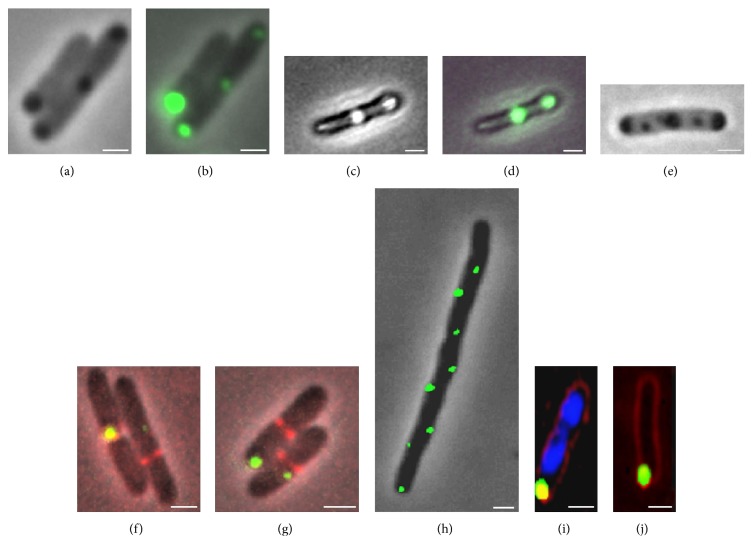
Accumulation of protein aggregates and inclusion bodies in *E. coli*. Reproduced with permission from Lindner et al. [180].

**Figure 2:**
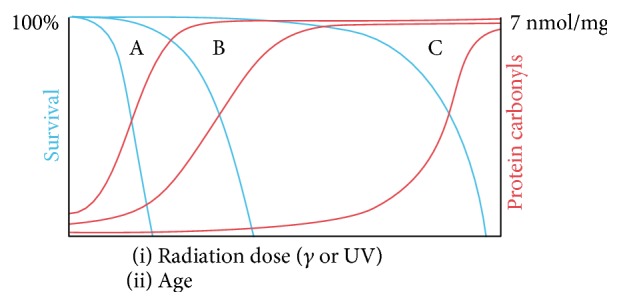
Schematic relationship between survival and protein carbonylation for different species (A, B, and C) as a function of radiation dose or age. Blue lines depict survival. Red lines depict protein carbonyl levels. Reproduced with permission from Radman [127].

The observations of senescence in unicellular organisms in the absence of genetic or environmental variability opened the door to suggestions that such organisms could be used as simple quantitative experimental systems to address molecular mechanisms underlying aging [79, 110]. Bacterial aging seems to share some common features with the process of eukaryotic aging, namely, the role of oxidative damage, and the effect of protein quality control systems to trigger senescence [166]. For instance, as in eukaryotes, bacterial aging is associated with the accumulation of oxidized proteins in the form of aggregates in the older poles of cells [76, 78] (Figure 1). This accumulation resembles many known age-related eukaryotic protein folding diseases [130, 131], and at least in eukaryotes, increased protein aggregation and altered cell proteostasis have been associated with oxidative stress-related posttranslational modifications [167]. Whether this process also plays a role in the accumulation of protein aggregates in bacteria remains unclear. The patterns of oxidative protein damage and aggregation accompanying aging in *E. coli* seem to be similar to those induced by UVA radiation, suggesting that the same type of ROS may be involved in determining cellular damage under both processes [81, 82]. In fact, the similarity between the biological effects of radiation and aging is easily observed in survival curves plotted as a function of radiation dose or time: both display a “shoulder” indicative of negligible mortality followed by an exponential decay in survival with increasing radiation dose or age (Figure 2) [127]. Thus, bacteria are now being considered as useful model organisms in aging studies, particularly in understanding the effects of aging and aging-related stress on protein stability and function [168].

**Figure 3:**
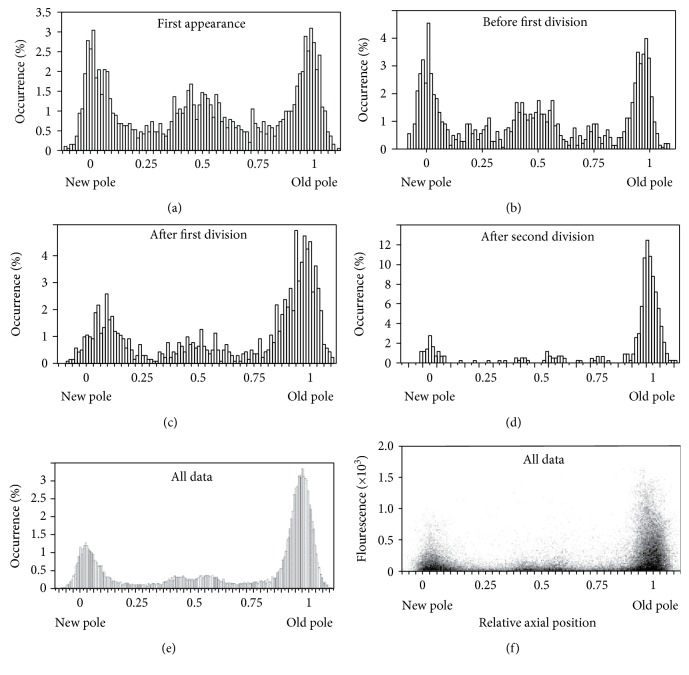
Aggregate distribution and associated fluorescence levels along the cell axis in *E. coli* throughout successive divisions. Reproduced with permission from Lindner et al. [180].

**Figure 4:**
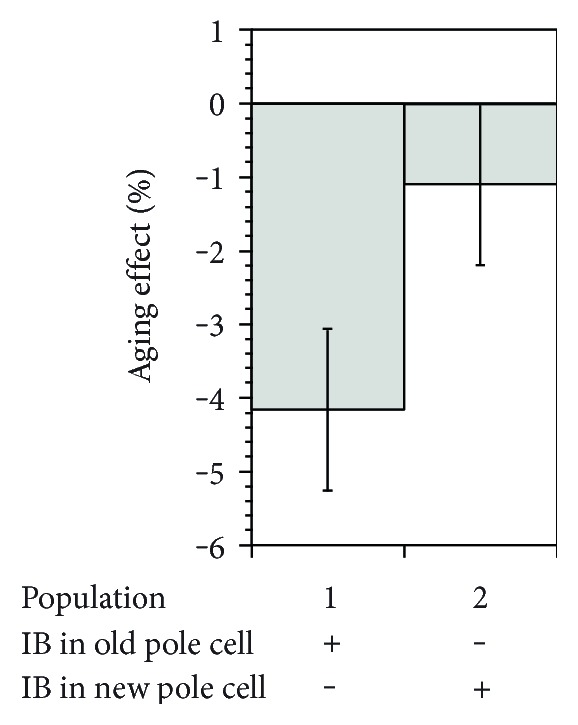
Aging correlation with the presence of protein aggregation. The aging effect was calculated from the relative growth rate difference between old-pole and new-pole offspring of newborn mother cells where inclusion bodies are inherited by the old pole cell (population 1) or the new pole cell (population 2). Reproduced with permission from Lindner et al. [180].

In the case of *E. coli*, a significant portion of the age-related fitness loss has been accounted for by the presence of protein aggregates that accumulate in the older bacterial poles (Figures 3 and 4). Misfolded proteins can passively and spontaneously aggregate at the cell poles in *E. coli* as a result of decreased diffusion and nucleoid occlusion [114, 118]. Thus, misfolded proteins freely diffuse in the cytoplasm and tend to stick to each other owing to the exposure of hydrophobic patches on their surface. As the amorphous aggregates grow by the addition of more misfolded peptides, they are excluded from the nucleoid and accumulate at the cell poles where they can expand further. Supporting this model, in silico simulations have demonstrated that the passive diffusion of a particle, its intrinsic ability to multimerize, and the absence of nucleoids at the poles are sufficient to obtain a polar localization pattern by entropy alone [169].

**Figure 5:**
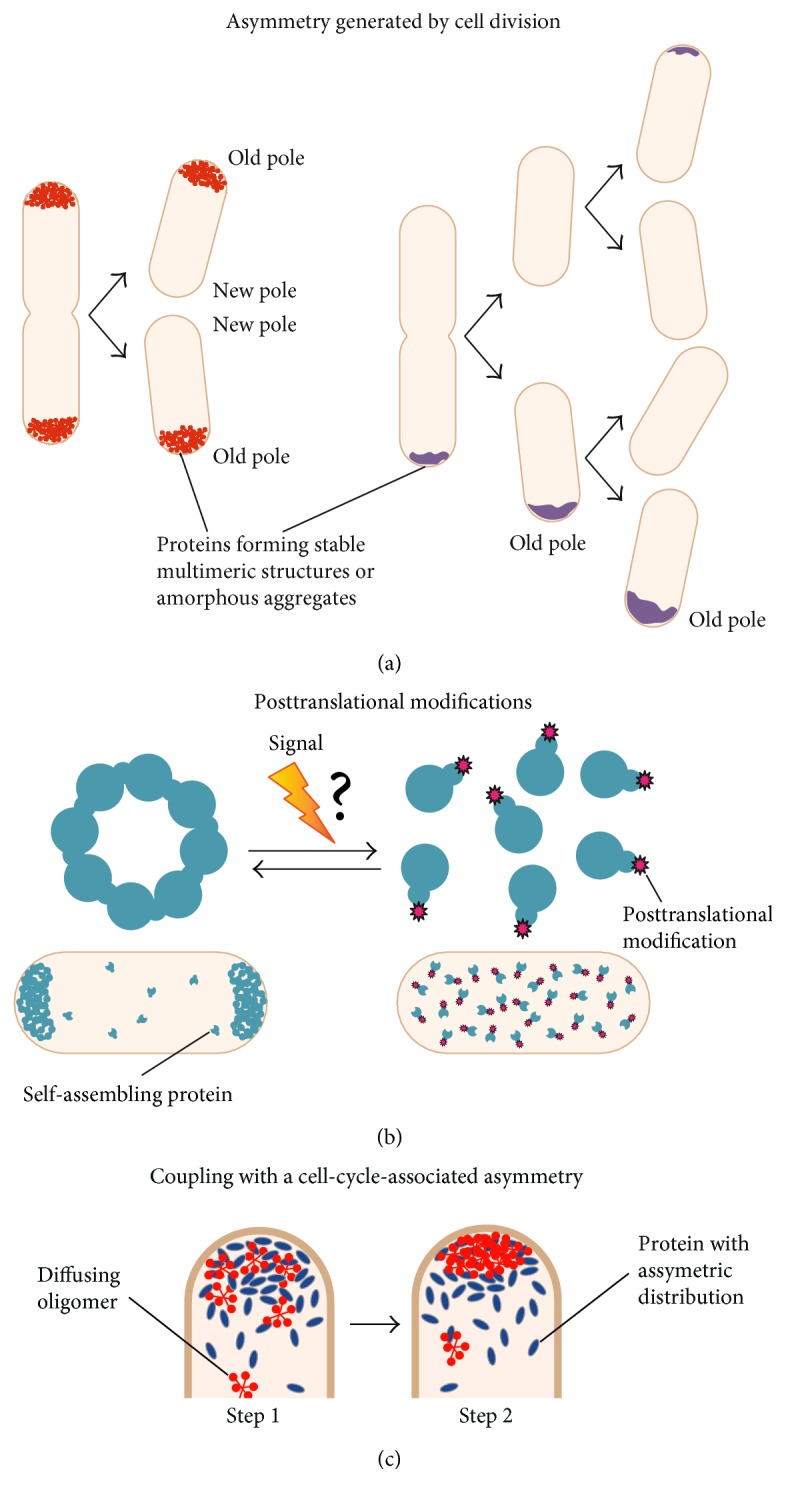
Possible strategies for spatial and temporal regulation of polar localization. (a) Asymmetric polar patterns can be naturally produced by a cell division event. Left: bipolar to old-pole localization; right: propagation of an old pole accumulation. Misfolded proteins produced in the progeny accumulate onto the existing polar aggregate. Eventually, de novo polar accretions can appear in progeny that did not acquire a polar focus (top cell), for example, after new protein synthesis. (b) The ability of some proteins to self-assemble and thereby to localize at the poles could be influenced by modifications such as phosphorylation upon a specific signal. The question mark indicates a hypothetical step. (c) The concentration of a self-assembling protein or oligomer and, thereby, its propensity to multimerize can be modified locally through protein-protein interaction with a partner whose subcellular distribution is asymmetric. In step 1, the protein (blue) has an asymmetric distribution inherent to a cell cycle event. The concentration of the diffusing protein oligomer (red) increases locally owing to interaction with the asymmetric protein. In step 2, the self-assembly of a protein or oligomer leads to the formation of a large structure at the pole. This provides spatial and temporal regulation to a multimerization-dependent polar localization. Reproduced with permission from Laloux and Jacobs-Wagner [169].

Additionally, a variety of posttranslational modifications, such as changes in phosphorylation state or nucleotide binding, can control the complex intracellular distribution of several proteins that are involved in cell cycle regulation, signal transduction, polarized motility, and adhesion [122, 170] (Figure 5(a)). Although most of the examples known to date are related to proteins that are at some point recruited to the poles through protein-protein interactions, similar modifications could also influence the ability of some proteins to multimerize, thereby impacting their spontaneous polar accumulation. If the presence and the activity of cognate kinases, such as phosphatases and GTPase-activating proteins (GAPs), is under the temporal regulation, this can provide a way to regulate an otherwise spontaneous polar localization in time (Figure 5(b)), as reported in the case of *Streptomyces coelicolor* [171–173]. Protein cleavage by specific proteases might also represent a strategy to modulate polar localization in space and time, as proposed for the polar beacon PodJ. PodJ is converted from a long form (PodJL) to a shorter form (PodJS) by a cell-cycle-regulated proteolytic sequence that eventually degrades PodJS, ensuring its proper localization and subsequently its function [123, 124] (Figure 5(c)). However, the precise mechanisms whereby both forms of PodJ differentially localize at the poles remain to be determined.

*N* ^ε^-Lysine acetylation has been recognized as a ubiquitous regulatory posttranslational modification that influences a variety of important biological processes in eukaryotic cells. Recently, acetylation has also been found to be prevalent among bacteria. Bacteria contain hundreds of acetylated proteins that affect diverse cellular pathways. Still, little is known about the regulation or biological relevance of nearly all of these modifications. To uncover the potential regulatory roles of acetylation, a recent study analyzed how acetylation patterns and abundances change between growth phases in *B. subtilis*. The authors discovered a subset of critical acetylation events that are temporally regulated during cell growth. Furthermore, they demonstrated a stationary-phase-enriched acetylation on the essential shape-determining protein MreB, which led them to propose a role for MreB acetylation in controlling cell width by restricting cell wall growth [174]. Lysine acetylation also coordinates carbon source utilization and metabolic flux in *Salmonella* in a reversible manner, so that cells are able to respond to environmental changes by promptly sensing cellular energy status and flexibly altering reaction rates or directions [175]. Thus, lysine acetylation may represent a metabolic regulatory mechanism that is conserved from bacteria to mammals. As evidence supporting the conservation of at least some of the hallmarks of aging in bacteria continues to emerge [74, 129, 176, 177], it will be interesting to investigate the role of PTMs in regulating bacterial aging.

## 6. Conclusions and Future Perspectives

As awareness of the role of PTMs in aging and aging-related diseases grows, there is an urgent need for the development of methods to detect protein PTMs more rapidly and accurately. Furthermore, the recent finding of rare and unconventional modifications in age-related pathologies calls for the development of more specific and sensitive methods to detect such modifications [27]. The recent rapid progress in large-scale genomics and proteomics technologies is likely to be a catalyzing factor for such studies. Drugs that target PTMs, such as phosphorylation, acetylation, methylation, and ubiquitination, will serve as useful tools in exploring the basic mechanism of PTM modulation and provide a pharmacological platform to combat the detrimental effects of aging [178].

From a nonpharmacological perspective, exercise interventions are known to be an effective means of delaying the negative effects of aging at the physical and metabolic level. Several lines of evidence have shown that exercise can bring about benefits for elderly people through the modulation of both inflammatory and redox status, with impacts on proteostasis, insulin sensitivity, body composition (e.g., adipose tissue, skeletal muscle) and hormonal profile, among others. Likewise, caloric restriction is another nongenetic and almost universal process known to delay the onset of aging and extend maximum lifespan [179]. However, the influence of exercise and diet on protein PTMs remains relatively underexplored. Studies covering this particular area have the potential to develop widely accessible and affordable intervention strategies to fight aging-related diseases.

Finally, the utility of prokaryotic models in understanding the biology of aging is noteworthy, given the possibility of the conservation of aging-associated molecular mechanisms throughout evolution. As research progresses in the field of microbiogerontology, it will be interesting to discover to what extent such molecular mechanisms are conserved. This might open a completely new window of opportunities to search for ways to slow aging and extend healthy lifespan.

## Figures and Tables

**Table 1:** Example of posttranslational modifications, their target amino acid residue(s), and the enzyme(s) or proteins involved [20, 77, 145].

Posttranslational modification	Target amino acid residue(s)	Enzyme(s) or proteins involved
Phosphorylation	Tyrosine, serine, threonine	Kinases, phosphatases
Glycosylation N-linked	Asparagine	Glycosyltransferases, deglycosylases
Glycosylation O-linked	Serine/threonine	Glycosyltransferases, deglycosylases
Acetylation	Lysine	Acetyltransferases, deacetylases
Methylation	Lysine, arginine	Methyltransferases, demethylases
Ubiquitination	Lysine	Ubiquitin-activating enzymes, ubiquitin-conjugating enzymes, ubiquitin ligases, deubiquitinases
Sumoylation	Lysine	Ubiquitin-activating enzymes, ubiquitin-conjugating enzymes, ubiquitin ligases, deubiquitinases
Myristoylation	Glycine	N-Myristoyltransferases
Prenylation	Cysteine	Farnesyltransferases, geranyl geranyltransferases
Palmitoylation	Cysteine	DHHC protein acyltransferases, acyl-protein thioesterases
Sulfation	Tyrosine	Sulfatases, desulfatases
S-Nitrosylation	Cysteine, methionine	
Glycation	Lysine	
Nitration	Tyrosine	Denitrases
Chlorination	Tyrosine	Myeloperoxidases
Oxidation/reduction	Cysteine	Peroxidases, oxidases, glutathione, thioredoxin
Carbonylation	Lysine, proline, arginine, threonine	
